# Editorial: Marine-derived food bioactives: understanding the impact of origin and extraction methodology on food quality, preservation and nutritional value

**DOI:** 10.3389/fnut.2024.1414325

**Published:** 2024-04-29

**Authors:** Joaquina Pinheiro, Narcisa Bandarra

**Affiliations:** ^1^MARE—Marine and Environmental Sciences Centre/ARNET—Aquatic Research Network, ESTM—School of Tourism and Maritime Technology, Polytechnic of Leiria, Peniche, Portugal; ^2^DivAV—Aquaculture, Upgrading and Bioprospection Division, IPMA—Portuguese Institute for Sea and Atmosphere, Algés, Portugal

**Keywords:** antioxidants, marine resources, extraction methods, marine-derived bioactives, antimicrobial, functional food, shelf-life, preservative

This Research Topic aims to address the effectiveness of conventional vs. emergent extraction methodology of marine-derived bioactive and investigate their quality, nutritional value, and potential preservation effect on food. One opinion and three papers covering the above-mentioned aspects are included in this Research Topic.

Salindeho et al. gathered the latest research about fish scales as a functional food that can help overcome child undernutrition, a global health problem that increases susceptibility to illness and fatalities. They found that 45% of child deaths are related to acute malnutrition and childhood illnesses such as diarrhea, pneumonia, and malaria and have severe short-, medium-, and long-term implications. Fish scales, regarded as by-products, have valuable components such as hydroxyapatite, collagen, and chitin, which can be utilized to produce bioactive peptides with various health benefits. Fish-derived peptides have different biological activities and can act as health promoters. Fish scales have the potential to act as a nutraceutical that can contribute for wellbeing and the prevention of disease.

In a study conducted by Lopez-Santamarina et al., the marine crustacean *Talitrus saltator* was analyzed to determine its nutritional value, heavy metal content, and potential to impact human gut bacteria. The findings were significant. This crustacean meets the nutritional criteria set by European regulation such as “high in protein,” “low in fat and sugars” and a good “source of fiber,” It also does not contain harmful levels of heavy metals but does have essential minerals like zinc, copper, and iron in amounts that meet daily requirements. The researchers also tested how *T. saltator* affected human gut bacteria *in vitro* and found some positive effects, like maintaining populations of healthy bacteria and stimulating specific metabolic pathways. However, they also found that it caused an increase in some potentially harmful bacteria, likely due to the presence of chitin, a substance in its composition that has antimicrobial properties. These findings underscore the need for more research better to understand the effects of *T. saltator* on gut health, and to explore its potential as a beneficial food source fully.

*Porphyra dentata* is a type of red seaweed that is highly nutritious and edible. It is a widely cultivated and consumed food in East Asia and has significant economic benefits. Recent studies have revealed that *P. dentata* is rich in bioactive substances, making it a potential natural resource with many benefits. In a recent study, a label-free shotgun proteomics technique was used to identify and analyze different harvest proteins in *P. dentata*. This technique shed light on a total of 13,046 different peptides and 419 co-expression target proteins that were characterized using bioinformatics analysis. The study performed by Yang et al. focused on protein characteristics, functional expression, and interaction of two critical functional annotations, amino acid, and carbohydrate metabolism. The results suggest that the bioactive peptides found in *P. dentata* may be utilized as high-quality active fermentation substances and potential targets for drug production. This research is noteworthy in that it integrates the global protein database, providing a framework for future studies based on a comprehensive understanding of the *P. dentata* proteome during different harvest periods. Overall, this study contributes to the construction of an information database and improves our understanding of *P. dentata'*s potential for use in various industries, inspiring new possibilities and biotechnological applications.

The research developed by Ren et al. aimed to explore the potential of low-molecular-weight peptides from monkfish (*Lophius litulon*) roe (known as MRP) to activate the immune system of immunosuppressed mice induced by cyclophosphamide (CTX). The results showed that when administered at a dosage of 100 mg/kg/d BW, MRP was able to improve the immune organ index, body weight, and morphology of the spleen and thymus in mice. Moreover, MRP was found to increase the levels of important immune markers such as interleukin (IL)-6, IL-1β, tumor necrosis factor (TNF)-α, and immunoglobulin (Ig) A, IgM, and IgG. Additionally, MRP could also mitigate the oxidative stress caused by CTX and activate the NF-κB and MAPK pathways in the spleen. These results suggest that MRP has excellent potential as an immune adjuvant or functional food for immunosuppressed individuals, offering a promising avenue for therapeutic interventions.

In summary, the studies and reviews mentioned above provide an overwhelming contribution of new and relevant data on marine resources, with important benefits for consumers. While there is existing literature and evidence on this critical topic, the papers in this Research Topic clearly show that many aspects of marine resources still need to be clarified and understood. Reading this Research Topic will, without a doubt, provide the reader with a clearer knowledge of topics such as the importance of understanding the biochemical composition, the most effective methodology for extraction, and biotechnological applications of marine-bioactive ingredients that successfully contribute to the improvement of human nutrition, prevention of disease and wellbeing of Society.

## Author contributions

JP: Writing – original draft, Writing – review & editing. NB: Writing – original draft, Writing – review & editing.

